# To 'take their place among the productive members of society': Vocational rehabilitation of WWI wounded at Erskine

**DOI:** 10.12688/wellcomeopenres.10581.1

**Published:** 2017-01-17

**Authors:** Jennifer Novotny

**Affiliations:** 1 University of Glasgow, Glasgow, Scotland, UK

**Keywords:** First World War, disability history, history of medicine, Scotland

## Abstract

In 1916, the foundation of the Princess Louise Scottish Hospital for Limbless Sailors and Soldiers (still in existence today as Erskine), on the banks of the River Clyde in Scotland, was a direct response to the need for specialised medical facilities to deal with the unprecedented number of injured service personnel returning from the Great War. At the hospital, the West of Scotland medical and industrial communities came together to mend broken bodies with prosthetic technology, as well as physical and mental rehabilitation to prepare the limbless to re-enter the job market. This paper explores the establishment of manual therapy workshops at Erskine and how such programmes of vocational rehabilitation were culturally informed by the concerns and anxieties of both the military and civilian populations of the First World War-era.

## Methodology

This paper is the result of archival research with a social historical approach, considering primary sources in the Erskine collection, as well as considering the materiality of health and wellness, taking into account the physicality of the vocational rehabilitation spaces from photographic evidence. The Erskine collection was recently acquired by the University of Glasgow Archives and Special Collections. It was made publicly accessible in 2016 after cataloguing and conservation funded by the Wellcome Trust Research Resources scheme.

## Introduction

By early 1916, with no cessation of hostilities in sight and mounting casualties, the abundance of limbless soldiers requiring treatment overwhelmed extant treatment facilities, such as Britain's main limbless hospital, the Queen Mary Auxiliary Hospital at Roehampton. From 1914 to 1918, over 40,000 British servicemen lost one or more limbs ( Anderson, 2011; pp45). Limbless patients required long months of aftercare as stumps healed, delaying the fitting of prosthetics, while some returning soldiers were so severely disabled that they required lifelong medical care. This exceeded the capacity of the military hospital network, which sought to deal with wounded quickly and efficiently and return them to frontline service. In the extreme press for beds, limbless servicemen unable to return to service could be discharged before proper healing, resulting in ill-fitting prosthetics and great personal discomfort. By 1916 the British public mobilised substantial charitable action to appropriately care for those permanently disabled by the war, and the foundation of Erskine was part of this response. By the end of the war, there were 6,000 registered charities specifically dealing with war disabled and these private charitable endeavours by far delivered the vast majority of long-term care and rehabilitation to disabled servicemen ( Cohen, 2001; pp35, 29). These charitable responses to the public health crisis engendered by the First World War were shaped by the cultural constructs of WWI-era society, which struggled to negotiate traditionally established definitions of masculinity and economic productiveness when faced with high numbers of wounded war veterans.

## Broken bodies

**Figure 1.**
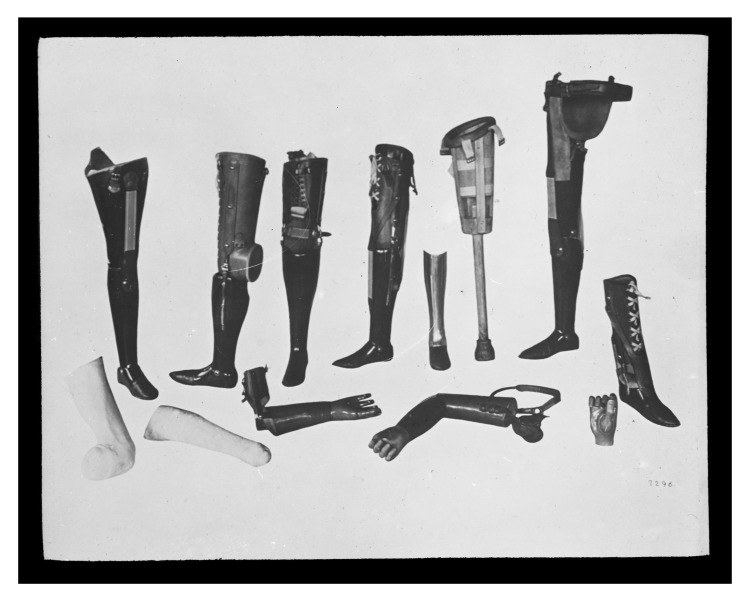
Examples of prosthetic limbs. University of Glasgow Archives reference DC079/173.

Broken bodies have always been the products of war, and British society had witnessed the physical consequences of conflict in the form of the disabled veterans of the Napoleonic Wars, the Crimea, and the South African wars. The mobilisation of vast numbers of volunteers and later conscripts alongside Regulars in WWI dramatically expanded the first-hand experience of war. Limblessness in particular was highly visible and required a wide variety of prostheses ( Figure 1). Empty sleeves and trouser legs were absences articulating not only the physical loss of part of the body, but notably for this study, also the loss of economic potential. Quite early in the war, each of the combatant nations (assuming their own victory, of course) showed a preoccupation with readying for a post-war economic boom. From the start, there were questions of how to best physically and economically rehabilitate war disabled so that they could be productive members of post-war society. Economic independence was not merely necessary for the financial viability of post-war governments, but also important to culturally constructed concepts of masculinity ( Bourke, 2016).

**Figure 2.**
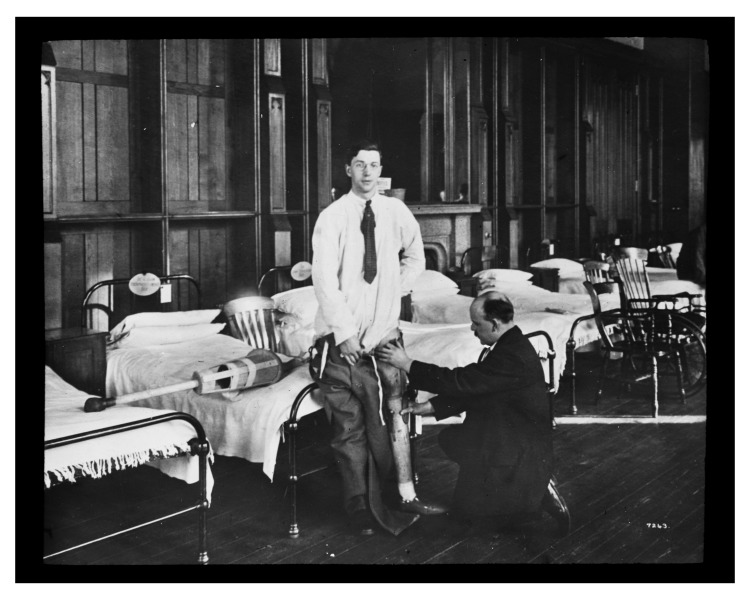
Fitting of prosthetic leg at Erskine. University of Glasgow Archives reference DC079/173.

As Julie Anderson (2011; pp 2) observes, the idea of ‘make do and mend’, popularised during WWII, could easily be applied to 20th-century attitudes toward disabled veterans of conflict: with proper rehabilitation '... seriously injured and permanently disabled servicemen could be reconstructed and reused in different contexts'. Thus, while the many men who listed their occupations in Erskine's patient intake register as labourers, miners, riggers, or shipwrights would not be able to return to their pre-war work, they could be retrained to serve some other capacity. Like a machine, the body could be repaired or otherwise repurposed with prostheses fitted like spare parts ( Figure 2) to suit modern industrial society.

The use of occupational therapy was developing prior to the First World War alongside the growing movement to define orthopaedic surgery as a medical specialization ( Perry, 2014). Doctors who specialised in the mechanics of the body saw the benefits of recuperative exercises, which included practical movements. There were good reasons to develop such therapies. Prior to 1914 there was a need in the civilian community to treat and rehabilitate individuals injured in industrial accidents. Employment programmes for ex-service personnel were also well established, with the Soldiers' and Sailors' Help Society offering programmes in 1885 and the Lord Roberts Workshops of sheltered employment open in 1904. The numbers and severity of injuries seen in WWI spurred the development of these trends - orthopaedics, occupational therapies, and employment for veterans - onwards at a quicker pace.

Popular pamphlets, printed lectures, and other publications suggest an ambiguous relationship with the new vocational therapies during the war years. Proponents asserted the importance of manual therapy “not just for exercising maimed limbs, but also for its ‘psychic’ healing effects” (qtd. in Perry, 2014; pp35). Other proponents ( McMurtrie, 1919; pp 40) were concerned about the nature of manual therapies and their suitability: ‘It is hardly fair to keep a man knitting when he may as advantageously take up some more masculine and practical occupation.’ Meanwhile others worried about the impact to the protected skilled labour market; influential surgeon Sir GT Beatson (1920; pp7) attempted to assuage these concerns, stating ‘[t]o prevent mistake it must be borne in mind that such workshops are not with the object of the occupational re-education of the wounded man, and they are not on the same basis as institutions where men are instructed so as to follow definite occupations.’ Thus, though it was widely agreed that giving disabled servicemen training in trades was beneficial, there were anxieties about what training was suitable and how exactly a re-trained individual should fit into the labour market.

## To 'take their place among the productive members of society'

The establishment of a specialist limbless hospital in the west of Scotland was championed by the pioneering surgeon Sir William Macewen (Honorary Surgeon to the King, Surgeon to the Admiralty, and Regius Professor of Surgery at the University of Glasgow). Macewen, along with John Reid, who purchased Erskine House and the adjoining property for the hospital, and industrialists like Harold Yarrow, of Yarrow's Shipbuilding Ltd. (Glasgow), launched a public appeal for funds for the new hospital on 29 March 1916, and by 10 April subscriptions totalled over £44,000. The hospital began to admit patients on 10 October 1916 with the capacity of 200 beds, which would double by 1917 (10/1/1917: pp130 ^1^).

**Figure 3.**
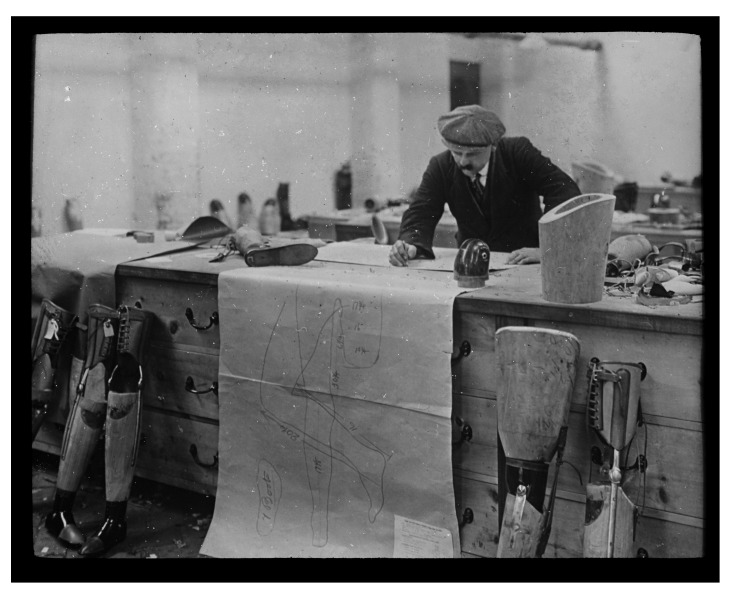
Designing artificial limbs. Engineers from the Clydeside shipyards were integral in the design process. University of Glasgow Archives reference DC079/173.

The hospital specialised in the aftercare of amputations. Re-amputating and surgically correcting previous operations was common, as attested by the operations books kept by Erskine’s surgeons. Macewen and the hospital’s founders fully recognised the need to provide lifelong medical care to amputees long after their stumps healed and prosthetics were fitted. In addition, the hospital actively undertook research into the improvement of prosthetic technology. Engineers from the Clydeside shipyards were integral in the on-going design process ( Figure 3).

Manual therapy workshops were conceived as a necessary part of the hospital from the outset. Early in April 1916 the Training for Employment Committee was established in order to 'arrange for such training and tuition as may be necessary or desirable for training and instructing the patients, with the view of their taking up certain trades, or entering into employment' (7/4/1916: pp9).

'For the patients at Erskine, the workshops were primarily to be used as an adjunct to the development of the function of the stumps and artificial limbs,' recorded the Sub-Executive Committee at its meeting in June 1916 (30/6/1916; pp61), '- for testing both and experimenting so as to secure the best form of instruments to enable the maimed to carry on the special kind of employment chosen - and incidentally to show the men how they may earn their own livelihood and take their place among the productive members of society.' This was all done under the watchful supervision of medical staff, to ensure the wellbeing of patients, who were not discharged until doctors felt healing and mastery of the use of new limbs had been achieved. There was no rush to clear beds, instead a programme of building modified and created new spaces for long-term residence. While many patients were fitted with limbs and discharged in a matter of months, some of Erskine's patients stayed on permanently at the hospital, working in various capacities.

### Work spaces

Upon acquiring Erskine House, the hospital directors worked quickly to adapt the early-19th-century stately home into a modern medical facility. Structural adaptations were necessary, such as the installation of a lift, new industrial boilers, increased power supply, and sewage lines.

**Figure 4.**
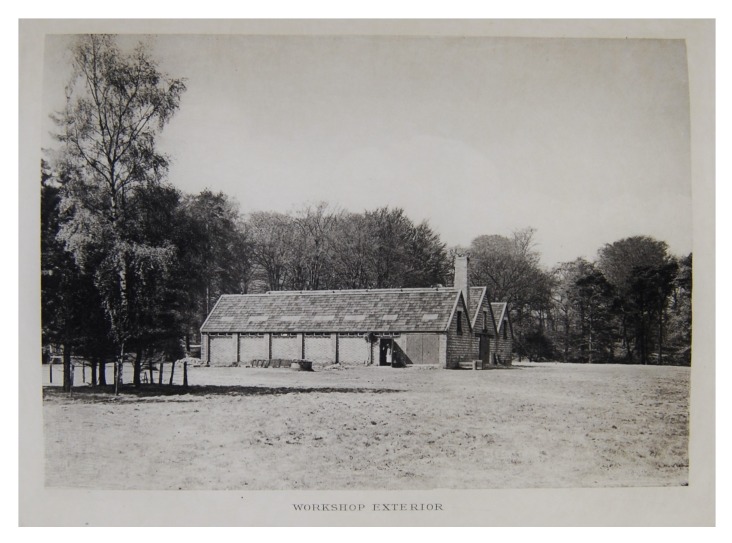
Workshops at Erskine converted from agricultural buildings. University of Glasgow Archives reference UGC225/10.

Workshops were outfitted in agricultural outbuildings ( Figure 4), converting stables, wiring them for machinery and adding heating (10/8/1916: pp70; 22/8/1916: pp76), as well as the establishment of a dedicated limb making shop adjacent to general shop (30/8/1916: pp80). Agricultural courses (poultry and pig keeping, gardening, dairying) were conducted in the extensive gardens and fields on the property. Inspiration was taken from other hospitals, specifically the 'mechano-therapeutics used at the Anglo-Belgian Hospital' (27/6/1916: pp56) and other facilities on the Continent. Strong links were maintained with similar facilities throughout the UK, sharing expertise with hospitals, such as the Ulster Volunteer Force Hospital at Queen's University Belfast, Ireland.

**Figure 5.**
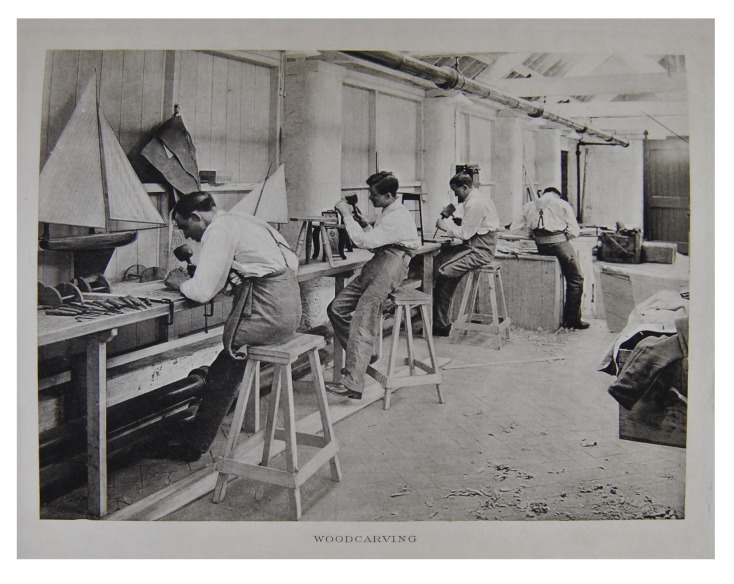
Woodcarving workshop. University of Glasgow Archives reference UGC225/10.

Lists of donations from members of the public are extensive: the civilian community responded to the health crisis caused by the war with overwhelming generosity, compensating for the limited support of official governmental bodies, such as the Ministry of Pensions. Subscribers had a stake in the hospital and were entitled to personally inspect the facilities at open days, as well as receive annual reports. There is a clear importance to early benefactors and patrons of the hospital in providing charitable support not simply through monetary donations, but by providing a variety of services and goods, as well as money earmarked for very specific items. For the workshops, sets of tools and machinery were purchased, donated or loaned. For example, a Mrs. King of Rozelle gifted the hospital a set of carving tools for the woodworking shop ( Figure 5) used by her late husband, who himself had been paralysed (27/6/1916: pp56), while additional sets of tools and a mitreing machine were purchased by the hospital with expert advice from local tradesman.

Likewise, it was attractive to contemporaries to support the hospital by purchasing items produced by the workshops. The workshops' later 1950s advertising slogan of 'help yourself by helping Erskine,' exhibits the idea of a mutually beneficial economic transaction: the purchaser gets something that they need, in addition to supporting a good cause. In the early years of the hospital, the workshops struggled to keep up with the demand for their products. Written requests for orders were routinely turned down in 1917 until production became well established. There was clear social cachet in supporting war disabled via this type of economic exchange. For example, Julie Anderson (2011; pp51) notes that items produced by the blind servicemen at facilities like St Dunstan's (today Blind Veterans UK) were popular because they '...allowed people to "consume" the spirit of heroic blindness'. Products made by war disabled, even quotidian items like baskets, offered a direct and tangible link to heroic sacrifice. They were physical reminders of a soldier's or sailor's devotion to duty, as well as enduring proof of the purchaser's charitable act.

By January 1917 the first workshops were fully operational with woodcarving, joinery and basket weaving on offer, and the addition of hand-loom weaving to be put in place with machinery loaned by a commercial weaver [Ross of Glasgow]. A memorandum was issued to patients at beginning of March 1917 (qtd. in minutes 6/3/1917; pp163), establishing the pay rates, hours, and training offered, highlighting the following objectives:

1. To enable a man to practise the use of his artificial limb in actual work before he leaves the hospital and the doctor’s care.
2. To enable the patients to assist the doctors and limb makers by suggestions of improvements in the limbs provided. (Prizes may be offered for best suggestions within a certain period.)
3. To give preliminary tuition in a new trade which a patient may select to follow, so that he may begin wage-earning on a higher scale on leaving hospital, or in an industry which may be carried on in leisure hours at home to augment earning or pension.

**Figure 6.**
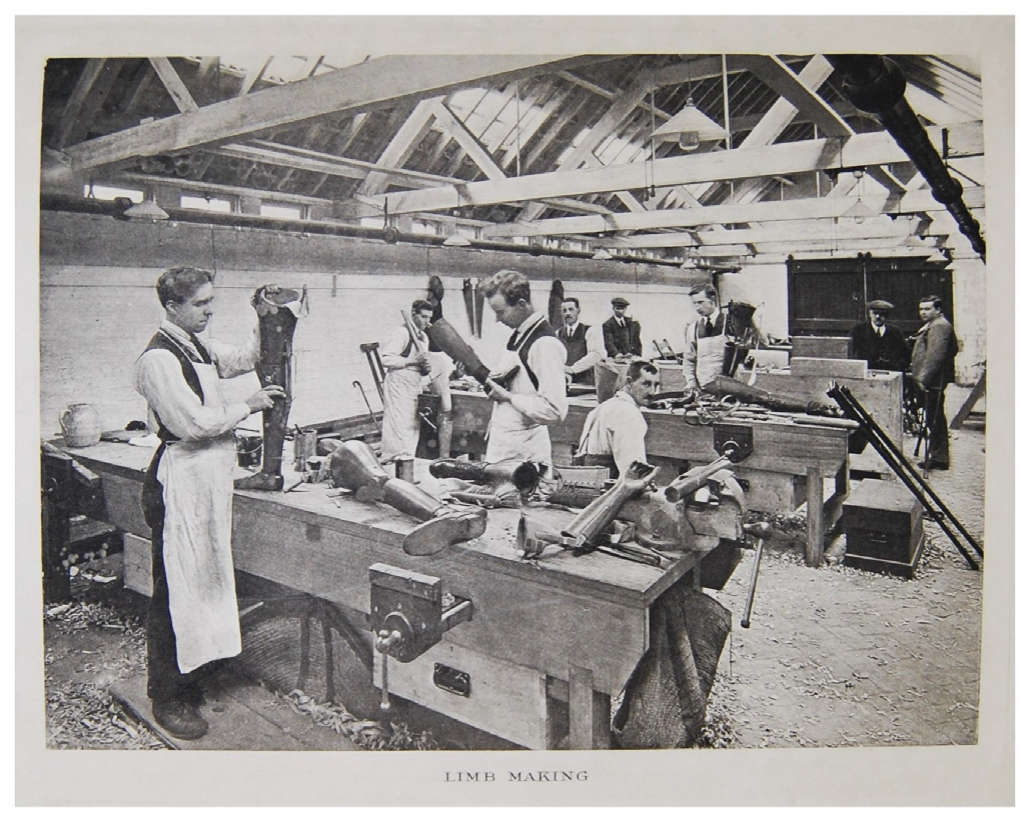
Limb making workshop. University of Glasgow Archives reference UGC225/10.

First and foremost is the practical, therapeutic aspect of training, followed by the technical development of prosthetics with patients actively encouraged to suggest improvements to design and functionality. Like many other limbless facilities during WWI, patients also directly contributed to the actual manufacture of the limbs in the on-site workshop ( Figure 6).

Working hours at the workshops were set as 10.00–12.00 and 14.00–16.00 on weekdays, as well as 10.00–12.00 on Saturdays. Full programmes of training at Erskine were designed to last 18–24 months, though this later came into conflict with regulations set by the Ministry of Pensions, which put an upper limit of 12 months on its approved training schemes. The Ministry of Pensions was adamant that such training should be "serious vocational training, not merely an occupation to help the man to get through the time" (27/11/1917: pp93). Furthermore, Ministry guidelines stated that "the workshops must be as well equipped as those of a technical school with competent instructors, and must be such as will satisfy a competent representative of the trade that the man is really going to be taught his trade effectively" ( *ibid*.), a requirement that Erskine initially found difficult to meet, though it did invest capital in augmenting and improving the workshops throughout 1917.

Attracting trainees was a challenge. Hospital authorities and the Ministry of Pensions had to repeatedly reassure disabled veterans that any income gained through training and subsequent employment would not adversely affect their pensions. Hospital authorities tried persuade patients to participate in the programmes by playing on fears that the low-wage jobs so readily available in 1917 (which often were taken up by war disabled) would not be so plentiful after the war, as well as appealing to a sense of patriotic duty. 'In acquiring a useful trade,' stated the Marquis of Ailsa, convener of the hospital’s training committee, 'a man is not only enhancing his own worth as an individual and his monetary work as a wage-earner, he is also rendering valuable assistance towards the commercial supremacy of his country by continuing to do his “bit” in the direction of filling our markets with goods of British manufacture to the exclusion of foreign-made articles.' (6/3/1917: pp164) Thus the disabled soldiers and sailors being fitted with limbs at Erskine were still being asked to ‘do their bit’, with economic rehabilitation couched in the terms of duty and service, much like the military service that had rendered them limbless; though they were no longer on the frontlines, they were still asked to serve.

There was also the incentive of direct compensation for work, subsidised by the government, which was payable upon discharge. In addition to this, the proceeds of the sale of goods manufactured in the training workshops would be split equally between the hospital and the patient (again, issued on discharge), less the cost of materials. There were bonuses for full completion of a course, as well as the accrual of a weekly wage. Those making saleable items were to receive part of profits (6/3/1917: pp164).

## Discussion

**Figure 7.**
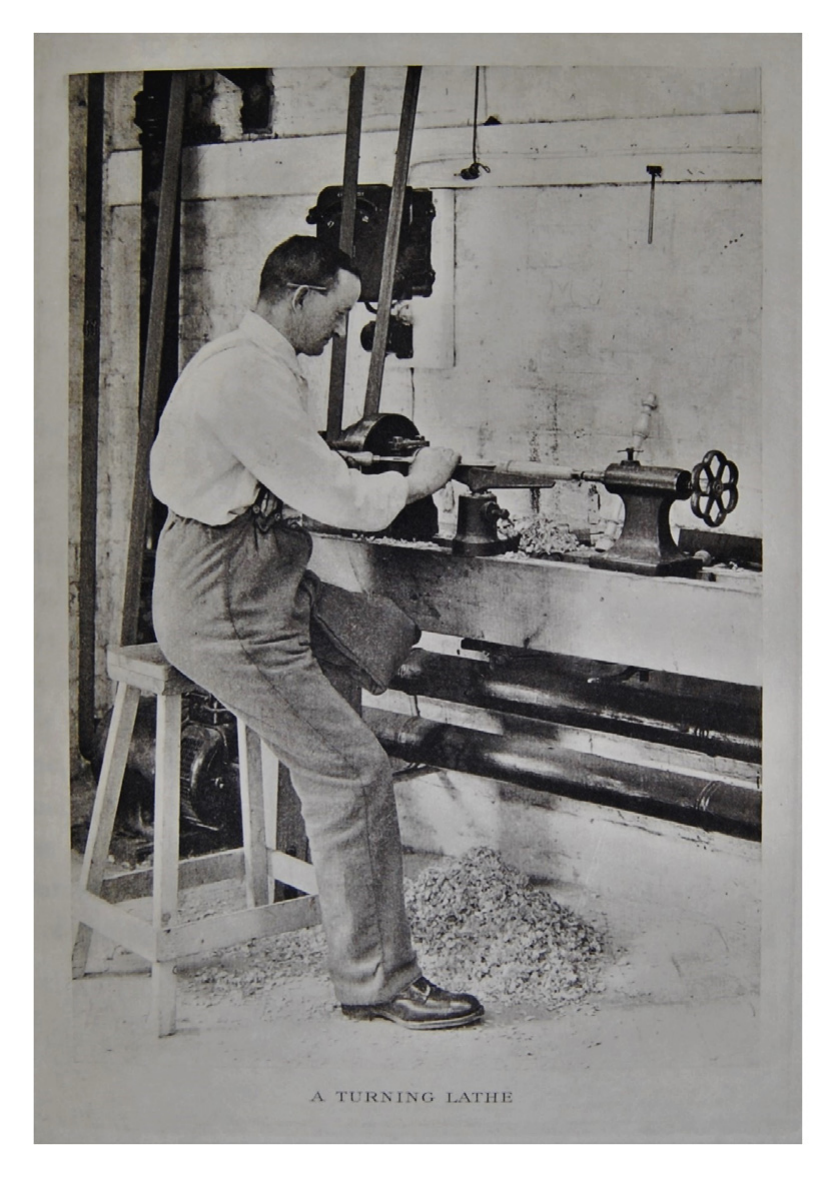
Patient at work. University of Glasgow Archives reference UGC225/10.

Workshops provided an alternative environment of healing outside of the hospital wards ( Figure 7), where a disabled serviceman could learn or enhance trade skills, socialise, earn extra money, alleviate boredom, exercise muscles and get used to new prosthetics. Manual rehabilitation facilities and training programmes were conceived, designed, and constructed by medical professionals and civilian benefactors of a certain social class, who had very specific ideas of masculine socio-economic roles and how disabled servicemen should adapt (and have their maimed bodies adapted) to them. The physical and psychological benefits of work were readily apparent to hospital authorities, and many men voluntarily participated in workshop training schemes, some of whom stayed in sheltered workshop employment (either at Erskine or elsewhere) for years to come. Many more, however, did not.

While some men undoubtedly benefitted from employment training, there were frequent complaints at Erskine, and more widely at national levels, of the lack of men taking up training, suggesting that not all war disabled were keen to participate. ‘…[T]he number of men engaged in the workshops is rather unsatisfactory, and that some inducement will require to be offered to encourage the men to take up duty,’ observed the Erskine training committee (6/3/1917: pp164). Likewise, in 1917 Ministry of Pensions officials noted that only 15% of disabled servicemen participated in national training initiatives ( Anderson, 2011; pp47–8).

Coercive tactics were employed by hospital authorities in the early years, but neither the economic incentives nor the spaces themselves were successful at attracting men in large numbers. Authorities also played upon culturally constructed ideas of duty for both the recuperating wounded, who were expected to continue to ‘do their bit’ for the socio-economic wellbeing of the nation, as well as the civilian population, who had a duty of care to the men maimed in service of their country.

It seems that most men, if they could, preferred to take up employment away from the oversight of hospital regulations and procedures. Despite the relatively small uptake of training places by Erskine patients, however, it has to be noted that sheltered workshops at the hospital endured into the 21st century, with the closure of the hospital's last workshop (furniture) in 2012. For a number of individuals disabled in WWI, WWII and subsequent conflicts, the economic opportunities provided by Erskine were life changing and small-scale production at the facility was economically viable for nearly one hundred years.

Erskine’s longevity attests to the need for such facilities throughout the 20 ^th^ century and beyond. Understanding the history of the physical, social, and economic rehabilitation of war wounded and their re-entry into civilian society is relevant to the experiences of service personnel today, many of whom are treated and supported by institutions established during the First World War. Identifying the impact of cultural constructs upon treatment regimens and facilities in the past can inspire reflection upon how we view and treat disabled veterans today, allowing us to see how far we have come and how much farther we still need to go.

## Footnotes

1 All meeting minutes cited are from the 1916–17 volume from the Erskine archive, held by the University of Glasgow, UGC225/1/1/1.
