# Supplementary material for: To 'take their place among the productive members of society': Vocational rehabilitation of WWI wounded at Erskine
Source: Wellcome Open Res. 2017 Jan 17;2:5. [Version 1] doi: 10.12688/wellcomeopenres.10581.1 (PMC5341767; doi:10.12688/wellcomeopenres.10581.1)
Supplement: Peer review history

# Peer review: Referee response for version 1

**DOI:** 10.21956/wellcomeopenres.11403.r19533

**Published:** 2017-02-20

**Authors:** Fiona Reid

**Affiliations:** 1 School of Humanities and Social Sciences, University of South Wales, Pontypridd, UK

The Princess Louise Scottish Hospital for Limbless Sailors and Soldiers was ‘an alternative healing space’ for amputees in the West of Scotland during the First World War. This study of the hospital (now known simply as Erskine Hospital) draws upon the Erskine Collection at Glasgow University to provide a very specific case study which highlights wider issues surrounding disability and rehabilitation.

 This article is grounded in a very particular time and place but recognises the importance of both the pre-war context and the long-term consequences of the hospital’s work with wartime amputees. World War One was not a *tabula rasa*: men had lost limbs in previous wars and in the industrial accidents of the late nineteenth and early twentieth centuries. Moreover, centres specialising in the complicated after-care required by the limbless have remained important and current practitioners can learn from this early history.

 This article makes impressive use of a particular set of archives but also demonstrates the broader hinterland involved in the history of early twentieth-century care for war-wounded men. The role of private charity was paramount but this involved more than simply putting money in a tin or agreeing to an annual donation. Donors were able to inspect the hospital workshops – and their annual accounts – and some donations indicate a highly personal link with the institution and its work. The story of the widow donating her paralysed late-husband’s carving tools is a case in point.

 Masculinity, and the preservation of masculinity looms large. The empty sleeve or pinned-up trouser-leg denoted a real loss on account of the cultural and economic importance of the limb. As a result disabled men were encouraged to ‘do their bit’, to become economically self-sufficient with the help of prosthetic limbs and to re-enter the appropriately masculine workspace. The intricate connections between the first total war and the industrial complex are especially pertinent here. The men were maimed by the fire-power of modern technology and those same industrial processes then became part of the cure as engineers from the Clydeside shipyards played a crucial role in designing prosthetic limbs.

 Like all good articles this one poses further questions for research. Why were so many men reluctant to train in the sheltered workshops? The author suggests that hospital regulations and processes played some role in this reluctance but it would be useful to probe further, especially given that one of the purposes of this research is to inform current practice. Nevertheless, this article highlights the economic, cultural and medical practices of the Erskine Hospital in the period of the Great War, and the photographic illustrations are well-selected to allow us a glimpse into the well-ordered and highly-organised attempt to re-create modern masculinity.

I have read this submission. I believe that I have an appropriate level of expertise to confirm that it is of an acceptable scientific standard.

# Peer review: Referee response for version 1

**DOI:** 10.21956/wellcomeopenres.11403.r19527

**Published:** 2017-02-13

**Authors:** Jeffrey S. Reznick

**Affiliations:** 1 History of Medicine Division, US National Library of Medicine, National Institutes of Health (NIH), Bethesda, MD, USA

This is a fine case-study overall: well-justified and thoughtfully done. However, it's very current historiographical frame falls short and therefore requires attention, to include two seminal works which appeared long before those by Anderson and Perry, namely Koven's article Remembering and Dismemberment (1994) and Reznick's book Healing the Nation (2004), and upon which the former studies build very well. The inclusion of these works, and perhaps also earlier others by Roger Cooter and Mark Harrison, will enhance the placement of this article in the broader literature, and make it all the more the fine piece that it is.

I have read this submission. I believe that I have an appropriate level of expertise to confirm that it is of an acceptable scientific standard, however I have significant reservations, as outlined above.

# Peer review: Referee response for version 1

**DOI:** 10.21956/wellcomeopenres.11403.r19426

**Published:** 2017-01-30

**Authors:** Julie Anderson

**Affiliations:** 1 School of History, University of Kent, Canterbury, UK

This article examines the rehabilitation programmes established at Erksine Hospital in Scotland during the First World War. Erskine was the main location for the aftercare of amputation, limb fitting, and vocational therapy for those patients in Scotland.

 The research makes a contribution to regional studies in the history of medicine in Britain. There is little work done on First World War rehabilitation in regional contexts, so this article compliments the work published mainly on English hospitals. Furthermore, the article offers a unique insight into the programmes in a single hospital, which provides points of comparison between Erskine and other military hospitals around Britain.

 Anxieties, both civilian and military, which surrounding the fate of the war wounded are addressed in this article. The form of post-war employment and whether suitable training was offered concerned the authorities and the public. The creation of ‘productive’ disabled veterans was central to the rehabilitative process and was bound up with concerns about employable bodies and masculine identity.

 The article makes a real contribution in its examination of the role of vocational therapy in hospitals during the war. The wounded men were offered new forms of employment which suited their level of impairment. Similar to other hospitals, Erskine was not merely a locus of medical care – its remit was expanded to develop and manage vocational therapies. Training in mechanics and agriculture was undertaken at Erskine, and the public supported the hospital through donations of money and equipment to outfit the workshops, and maintained vital levels of support by buying items produced at the hospital. The article details Erskine’s longevity, supporting the notion that vocational units within hospitals provided a vital role in rehabilitation throughout the twentieth century. The paper makes the important point that policy makers, practitioners, and others currently responsible for disabled veteran’s support should reflect on the successful programmes established at Erskine over 100 years ago.

 Overall, this paper offers a fascinating insight into a relatively understudied war hospital. It is gratifying to see rich archival sources utilised to provide new and innovative research which assists our knowledge, understanding, and subsequent analysis of the range of therapeutic provision in hospitals in Britain during and after the First World War.

I have read this submission. I believe that I have an appropriate level of expertise to confirm that it is of an acceptable scientific standard.
